# The role of tubular cells in the pathogenesis of Fabry nephropathy

**DOI:** 10.3389/fcvm.2024.1386042

**Published:** 2024-04-05

**Authors:** Paula Rozenfeld, Sandro Feriozzi, Fabian Braun

**Affiliations:** ^1^ Instituto de Estudios Inmunológicos y Fisiopatológicos (IIFP), UNLP, CONICET, Asociado CIC PBA, Facultad de Ciencias Exactas, La Plata, Argentina; ^2^ Nephrology and Dialysis Unit, Belcolle Hospital, Viterbo, Italy; ^3^ III. Department of Medicine, University Medical Center Hamburg-Eppendorf, Hamburg, Germany; ^4^ Martin Zeitz Center for Rare Diseases, University Medical Center Hamburg-Eppendorf, Hamburg, Germany; ^5^ Hamburg Center for Kidney Health, University Medical Center Hamburg-Eppendorf, Hamburg, Germany

**Keywords:** Fabry disease, nephropathy, tubular cells, pathogenesis, fibrosis

## Abstract

The pathophysiology of Fabry nephropathy (FN) is induced by galactosidase A deficiency with a chronic exposure of glycolipids to every lineage of renal cells. Tissue damage is attributed to the activation of molecular pathways, resulting in tissue fibrosis and chronic kidney disease. Podocytes have been the primary focus in clinical pathophysiological research because of the striking accumulation of large glycolipid deposits observable in histology. Yet, the tubular interstitium makes up a large portion of the whole organ, and therefore, its role must be further considered in pathogenic processes. In this review, we would like to propose Fabry tubulopathy and its ensuing functional effects as the first pathological signs and contributing factors to the development of FN. We will summarize and discuss the current literature regarding the role of tubular cells in Fabry kidney pathophysiology. Starting from clinical and histological evidence, we will highlight the data from animal models and cell cultures outlining the pathophysiological pathways associated with tubular interstitial injury causing renal fibrosis in Fabry nephropathy.

## Introduction

Fabry disease (MIM 301500) is an X-linked lysosomal disorder caused by the deficient activity of the enzyme alfa galactosidase A (GLA) (EC:3.2.1.22), which leads to an intracellular deposition of complex sphingolipids caused by pathogenic variants in the *GLA* gene. The disease is phenotypically described in patients as “classic” or “late onset” (1). The kidney is one of the predominantly affected organs in Fabry disease. Patients with classic Fabry disease suffer from progressive kidney affection, resulting in mild proteinuria and a deterioration of renal function with the development of chronic renal failure. Patients with late-onset disease develop Fabry-associated organ damage mostly during adulthood, which is commonly restricted to one or two organ systems (2).

Structural changes involve all compartments of the kidney, including the tubular system and interstitium. The pathophysiology of Fabry nephropathy (FN) is attributed to tissue damage induced by GLA deficiency with a chronic exposure of globotriaosylceramide (Gb3) and its deacylated form globotriaosylsphingosine (LysoGb3) to cells and tissues. Every lineage of renal cells, glomerular epithelial cells (podocytes and parietal cells), and mesangial, vascular, and tubular cells show involvement in these pathways, leading to cellular decay and a chronic activation of inflammation, ultimately resulting in tissue fibrosis and chronic kidney disease (3).

Podocytes have been the primary focus in clinical research aiming to better understand the progressive renal failure associated with Fabry disease. Glycolipid deposits in podocytes are striking on Fabry kidney histology. Podocytes are long-lived, terminally differentiated cells with a limited capacity of replication (4) and separated from the bloodstream by the basement membrane (after birth) (5, 6). These features may be one of the bases for which these cells present with the highest amount of Gb3 inclusions and for the high resistance to the clearance of Gb3 in treated patients. Their dysfunction and loss are closely associated with glomerular injury, proteinuria, and declining renal function.

On the other hand, renal tubular cells are also affected by Fabry disease. The tubular–interstitial involvement attracts less attention perhaps because of mild clinical signs such as polyuria or urine concentration defects. The tubular interstitium makes up a large portion of the whole organ, and therefore, its role must be further considered in pathogenic processes (7). Tubular cells are dividing cells with a high metabolic expenditure and energy consumption. Their main function is the reabsorption of the bulk of the glomerular ultrafiltrated and secretion of solutes into the urine. Beyond this, they are among the cells with the highest number of mitochondria per cell in the body.

In this review, we would like to propose Fabry tubulopathy and its ensuing functional effects as the first pathological signs and contributing factors to the development of FN. We will summarize and discuss the current literature regarding the role of tubular cells in Fabry kidney pathophysiology. Starting from clinical and histological evidence, we will highlight the data from animal models and cell cultures outlining the pathophysiological pathways associated with tubular interstitial injury causing renal fibrosis in Fabry nephropathy.

## Clinical manifestations and histologic features

In males with the classic Fabry disease, signs of renal involvement appear during the second decade of life; patients develop mild proteinuria (<1 g/24 h) and a progressive reduction of renal function. End-stage renal disease occurs in most untreated patients around the age of 40. In the late-onset variant, patients have a less severe nephropathy (8, 9).

The intracellular deposition of Gb3 and LysoGb3, caused by the reduction of GLA activity, takes place in all lineages of renal cells. The histological changes in FN start early when clinical signs are absent or minimal. Tøndel et al. reported podocyte foot process effacement and intracellular Gb3 inclusions occurring very early in life. They occur when proteinuria is mild or even in the normal range (10, 11). Over time, podocyte injury leads to the detachment and loss of these cells into the urine, with consequent podocyturia (6, 12). In light microscopy, the visceral and parietal epithelium shows the typical, but not pathognomonic, aspect of vacuolized cells (foamy appearance), while glomerular tufts can depict focal segmental sclerosis. The mesangium expands and presents with hypercellularity. The vasculature can develop increased wall thickness with a hyperplasia of pericytes and smooth muscle, resulting in a remodeling of its structure (12). In tubular epithelial cells, there is a pronounced preference for deposition in the epithelium of the distal tract (10), which may also occur in proximal tubules (13).

The difference between histological levels of deposits between podocytes and tubules could be attributed to the distinct turnover rate of these cells. Podocytes are long-lived non-dividing, terminally differentiated cells that accumulate Gb3 throughout the organism's lifetime. In contrast, tubular cells are dividing cells, and as a consequence, lower deposition levels are found in kidney biopsies.

Trimarchi (14) recently demonstrated that proximal tubules are functionally affected by the decrease of megalin, cubilin, and electrogenic chloride/proton exchanger ClC-5, which may affect the activity of ClC-5, a Cl^−^/H^+^ antiporter channel mainly located in early endosomes where it is involved in the acidification process of lysosomes. Furthermore, a reduced expression of uromodulin, Na^+^-K^+^-ATPase, and Na^+^-K^+^-2Cl cotransporter in thick ascending limb cells has been demonstrated, which could be responsible for the occurrence of polyuria in patients with Fabry disease (15).

Tubular functional alterations are not extensively reported. This is attributed to the fact that the clinical symptoms or complaints related to these alterations are minimal and do not draw the attention of clinicians and patients. In addition, the tests to investigate tubular alterations are cumbersome and therefore are not commonly performed in nephrology units. Nevertheless, concentration defects and polyuria are commonly reported symptoms. This is underlined by the fact that fibrotic lesions such as tubular atrophy and interstitial fibrosis can be present starting from the early phases of FN (3). In more advanced stages of FN, glomeruli are obliterated by global sclerosis, and tubular atrophy is associated with interstitial fibrosis in different phases (8).

The effects of specific therapy on tubular epithelial cells have been minimally investigated. While we have enough data on the cellular clearance of Gb3 from glomerular and endothelial cells, limited data are available for tubular cells. In an early paper on the effect of agalsidase beta on cellular lipid inclusions, Thurberg et al. described the clearance of Gl-3 from distal tubular epithelial cells after 11 months of therapy was initiated in 50% of patients. This value is lower than expected, considering the high turnover of the tubular epithelial cells. We can speculate that specific treatment effectively reduces Gb3 deposition, but the clearance could not be complete (16–18).

Therefore, with regard to the tubular alterations in FN, we have a limited clinical description despite extensive histological evidence and the growing knowledge on the mechanisms by which tubular interstitial fibrosis often leads to end-stage renal disease.

## *In vitro* studies

*In vitro* studies have predominantly investigated the molecular pathomechanisms in Fabry podocytopathy. These delineated the direct effects of LysoGb3 on podocytes triggering a profibrotic effect through transforming growth factor (TGF)-β1 and Notch1 signaling with fibronectin and Collagen 4 deposition (19, 20). Likewise, Kim reported on decreased podocyte survival upon LysoGb3 treatment due to RIPK3-mediated necroptosis (21). Beyond this, GLA deficiency resulted in decreased mTORC1 activity with a subsequent increase in autophagy (22). To what extent these effects are solely dependent on Gb3 deposition was recently put into question, as Gb3 clearance of Fabry podocytes using enzyme replacement therapy failed to restore aberrant autophagy and Notch 1 signaling (23). This could also indicate that there is a point of no return in the pathogenesis, especially once profibrotic signaling is triggered. Another explanation for this can be substrate-independent mechanisms such as other lysosomal proteins accumulating and aggravating lysosomal dysfunction, such as the recently reported alpha-synuclein (24).

Jehn et al. (22) identified the acid sphingomyelinase ASAH1 to be upregulated in tubular cells of a patient with Fabry disease. This is particularly interesting, as decreased sphingomyelin, e.g., due to increased ASAH1 expression was detected as a feature of murine kidney aging. (25). In Fabry podocytes and patient-derived urinary cells, there was an increased abundance of lysosomal proteins under GLA-deficient conditions (26). These aberrations could be crucial, as lysosomes are implicated in multiple cellular processes affecting even the structure and function of the endoplasmic reticulum (ER) and mitochondria (27). Likewise, misfolded GLA proteins can trigger direct effects on cellular organelles such as ER stress (28, 29).

Tubular cells collected from the urine samples of patients with Fabry disease revealed an impairment of mitochondrial morphology and increased oxygen consumption rate reflecting mitochondrial dysfunction (30), a pathologic trait also reported in Fabry fibroblasts (31). In line with this, siRNA-mediated downregulation of GLA protein abundance in human proximal tubular cells (HK-2) leads to increased autophagy in a transcription factor EB (TFEB)-dependent manner, in turn, resulting in increased ROS production and proapoptotic signaling (32). In kidney organoids, GLA-KO leads to a decreased expression of multiple nephron markers with increased ROS production and decreased signals for mitochondria with the accumulation of intracellular calcium (33). As the tubular system of the kidney is highly dependent on mitochondrial function to uphold the transcellular transport of solutes and active secretion of compounds into the urine, a dysfunction of the central energy metabolism can have widespread effects. Gb3 and LysoGb3 exposure of HK-2 furthermore results in a wide range of transcriptional abnormalities indicative of epithelial-mesenchymal transition (EMT) (34) that is dependent on an increased expression of TGF-β, N-Cadherin, and alpha-smooth muscle actin (α-SMA) and phosphorylation of the PI3K/AKT pathway.

Overall, the current *in vitro* data point toward mitochondrial dysfunction as a central mechanism in Fabry tubulopathy, which could be the driving force behind profibrotic signaling and EMT through increased ROS production and decreased cellular metabolism.

## Animal models of Fabry disease

In the late 1990s, Ohshima et al. presented the first Fabry mouse model, GLA-KO, generated by deleting the *GLA* gene (35). Surprisingly, GLA-KO mice clinically exhibited a normal phenotype despite a progressive accumulation of Gb3 in the kidneys, ranging from 3 to 5 times the levels of the wild type (36). The failure to represent the phenotype of the full human disease can be explained by the fact that the metabolism of glycolipids differs in mice, with Gb3 levels being much higher in murine liver than in the kidneys, while the opposite relationship is observed in patients (37).

A histopathological analysis of a GLA-KO mouse kidney by hematoxylin–eosin staining revealed the presence of inclusions in proximal and distal tubular epithelial cells, and in the glomeruli, parietal epithelial cells revealed the highest amount of cytoplasmic inclusions with only small and inconspicuous podocyte inclusions (38). Strikingly, the electron microscopy of GLA-KO kidney tissue revealed electron-dense concentric lamellar structures only in tubular cells.

Since the limited development of the Fabry phenotype could be due to a lower capacity for synthesizing and accumulating Gb3 per tissue mass than humans, a symptomatic Fabry model mice was developed by cross-breeding the GLA-KO mice with a Gb3 synthase transgenic mice (GLA-KO-Tg). These mice presented higher Gb3 levels in serum and organs as compared to the GLA-KO mice. In addition, serum LysoGb3 was detected at higher levels in GLA-KO-Tg (39).

Electron microscopy revealed electron-dense concentric lamellar structures in the proximal and distal convoluted tubules and collecting ducts, with a small number of lipid inclusions in podocytes (15). The evaluation of renal function in GLA-KO-Tg revealed a deficiency of concentrated urine, the presence of albuminuria, and increased blood urea nitrogen. GLA-KO-Tg mice showed early lethality associated with the loss of body weight, neurological abnormalities, and progressive renal impairment characterized by polyuria, polydipsia, and decreased urine osmolality, which resulted in water- and salt-loss phenotypes, without remarkable glomerular damage (40). The treatment of these mice with recombinant agalsidase resulted in Gb3 clearance from organs, low serum lysoGb3 levels, and reduction in urine albumin concentration.

A decreased ability to concentrate urine, leading to polyuria, is thought to be the first symptom of Fabry disease (41) that could be attributed to distal tubular dysfunction. Moreover, albuminuria might be caused by decreased protein reabsorption at the proximal tubules. These findings indicate that the renal impairment seen in GLA-KO-Tg mice may correspond to the initial steps of renal involvement in patients with Fabry disease (42).

We could assume from this model that the first signs of kidney involvement mainly affect tubular cells instead of podocytes or other glomerular cells. In this model, tubular cells contained the most pronounced lamellar bodies, rounded mitochondria, and disorganized, flattened infoldings. On a molecular and histological level, the tubular–interstitium compartment showed signs of inflammation, oxidative stress, and focal fibrosis, alongside macrophage infiltration. As this pattern occurred without causing profound podocyte injury, it may be important to note the occurrence of tubule injury in addition to podocyte injury in human FN.

Further insights can be gained from a model of unilateral ureteral obstruction (UUO) in GLA-KO mice. This resulted in increased fibrosis and an increased number of tubular apoptotic cells, suggesting that Fabry disease is associated with enhanced tubular susceptibility to apoptosis (42).

The GLA-KO rat model further reinforces the proposition of tubular cell affection as an early sign of FN (43). Fabry rats developed proximal tubular disease that manifested as increased urine flow rate, decreased osmolarity, and increased urine calcium with age. A decline of the estimated glomerular filtration rate also occurred at a later stage. Functional analysis correlated to the histological data, with the proximal tubule cells of KO rats appearing more vacuolated with the accumulation of several large, circular inclusions.

Additional insights into the pathology of FN will definitely be shaped by animal models beyond rodent systems. The first evidence for this is presented through a zebrafish model lacking the GLA homolog. Despite missing Gb3 deposition, due to the fact that zebrafish do not express Gb3 synthase, the model developed increased creatinine levels and proteinuria, pointing toward mechanisms beyond substrate accumulation (44).

Although Fabry disease models to date have failed to represent the full Fabry disease classical phenotype as present in patients, they inform us that a minimal biochemical alteration associated with glycolipid deposits may have an impact on tubular kidney cells that could be related to the development of fibrosis. They reinforce the notion that exposure to low levels of Gb3 early in the disease can trigger a cascade of events. Moreover, of the many different cell types in the kidneys, the tubule could be the most affected one at low deposit levels and is responsible, at least in part, for the development of fibrosis.

## Pathogenetic pathways of fibrosis in nephropathies

Based on clinical and histological features, and taking into account animal and cell culture studies, we propose a decisive role for the tubular epithelium and interstitium in causing renal fibrosis in Fabry nephropathy.

Renal fibrosis involves the deposition of the extracellular matrix that determines glomerulosclerosis, vessel arteriolosclerosis, tubular atrophy, and interstitial fibrosis (45). In particular, it needs to be pointed out that the expansion of interstitial fibrosis is the best marker for the progression of renal disease, outperforming the assessment of glomerular damage (46).

Cell damage causes the activation of inflammatory processes with the infiltration and activation of immune cells: neutrophils, macrophages, and dendritic cells. Fibrogenesis results from a long-lasting activation of the pathways and a wound-healing response to tissue injury not resolving (47). This process is marked by inflammation, myofibroblast activation, migration, and matrix deposition (48). The activated immune cells and/or modified epithelial cells stimulate the release of profibrotic cytokines such as TGF-β, platelet- derived growth factor (PDGF), among others. Among the recruited cells, myofibroblasts are detected in the interstitium, the arterioles, and the mesangium. Kuppe et al. demonstrated that pericytes and fibroblasts are the primary cellular source of myofibroblasts (49). α-SMA is the marker of myofibroblast, and it produces filaments anchoring the myofibroblast to the matrix during the process of reorganization and healing (48). In Fabry nephropathy, Rozenfeld et al. demonstrated a pivotal role of myofibroblasts. On renal tissue from biopsies of Fabry patients, proximal tubular cells produce TGF-β, inducing the activation of myofibroblasts in the vessels and glomeruli, and stimulate tissue fibrosis (50).

A recent yet growing body of evidence suggests that these processes are, in part, propagated by an increased systemic inflammatory response. Early depiction of this phenotype reported dendritic cells and monocytes to be involved through direct Gb3 action via TLR4 (51). Both endogenous Gb3 and exogenous LysoGb3 can contribute to these effects (52). Beyond this, TNFa and MCP-1 were increased in patients with Fabry disease, coinciding with elevated CCR2 levels on monocytes (53). Furthermore, an involvement of T cells and an effect on their ratios upon enzyme replacement therapy (ERT) were shown (54, 55), while there were reports of decreased Il-4 production by invariant NKT cells (56). In whole peripheral blood mononuclear cells (PBMCs), differentially altered expressions of TNF and TLR4 were detected (57), and several studies showed higher IL6 and TNFa levels in patient sera (58, 59). The same reports, however, showed conflicting data on Il-1beta. Most recently, first evidence was presented on the therapy-resistant activation of the complement system, further indicating a systemic response (60). In solid tissue, increased monocyte adhesion to Fabry endothelial cells was shown, as also an improvement of this immune attraction through anti-inflammatory drugs (61).

Such chronic inflammation and dysregulated matrix production impair blood flow and cause reduced availability of oxygen to mitochondria. This hypoxia alters mitochondrial function with an increase in reactive oxygen species and collagen synthesis (62). As reported in the previous section, An et al. (32), who studied lysosomes, found significant alterations in number, energy, and fuel consumption. The levels of oxidative stress were high, and oxidative phosphorylation was upregulated. Disturbed fatty acid oxidation has already been linked to interstitial renal fibrosis (63).

TGF-β is a master regulator of the fibrotic process, and its production increases with chronic inflammation in chronic renal disease. In an *in vitro* model of Fabry nephropathy, Jeon et al. (64) demonstrated that under the exposition of Gb3, the human renal proximal tubular epithelial cells undergo EMT driven by TGF-β upregulation. Moreover, lasting damage results in a cell-cycle arrest of tubular cells with subsequent senescence and apoptosis, followed by tubular atrophy and interstitial fibrosis (65). TGF-β can also activate extracellular matrix protein deposition by podocytes *in vitro*. However, TGF-β production is not revealed in the glomeruli of kidney biopsies from patients with Fabry disease (50). This difference suggests that although podocytes are cells with the highest levels of Gb3 deposits, they may not be the ones that create a TGF-β-associated profibrotic environment.

The deposition of Gb3 in the Notch1 system seems to trigger the NF-kappa-B signaling pathway stimulating the synthesis of chemokine production (19, 20). There is evidence in the literature that the activation of Notch1 determines fibrosis through the activation of the EMT transcriptional program (3, 66).

Moreover, metalloproteinases (MMP) affect the process of fibrosis. MMP-7 can activate EMT, TGB-β signaling, and ultimately the deposition of the extracellular matrix (67). In tubular cell cultures from mice and patients with Fabry disease treated with Gb3 and LysoGb3, an upregulation of the metalloproteinase 9 (MMP9) gene has been demonstrated. MMP-9 degrades extracellular matrix proteins and activates cytokines and chemokines to regulate tissue remodeling (68).

Li et al. (69) reported that renal fibrosis starts focally in specific sites defined as “niches.” The niches include kidney residents, infiltrated cells, and the extracellular matrix. The niches have a specific microenvironment that activates fibroblasts. There is a progressive diffusion of fibrotic processes driven by a specialized network that includes tenascin C, connective tissue growth factor, fibrillin 1, and periostin, resulting in an activation of all significant steps causing tissue fibrosis.

The above processes determine fibrosis when they last over time and the disease progresses. However, the fibrosis seems potentially reversible, and the interstitial matrix can resolve renal fibrosis (70). Some evidence indicates that macrophages, ADAMTS13, metalloproteinase, and other proteins can be exploited to tip the balance between production and degradation of the extracellular matrix toward resolution (71).

Lastly, a significant factor further aggravating tubulointerstitial fibrosis occurs as soon as glomerular damage leads to constant (micro-)albuminuria. Currently, our pathophysiologic understanding still points toward a role for albumin as a mediator of tubular damage and renal fibrosis (72, 73). Therefore, future research efforts need to focus on both the clinical and the histological benefits that novel strategies of lowering albuminuria and renal protection bring to patients with Fabry disease (74).

## Conclusions

**Figure 1:**
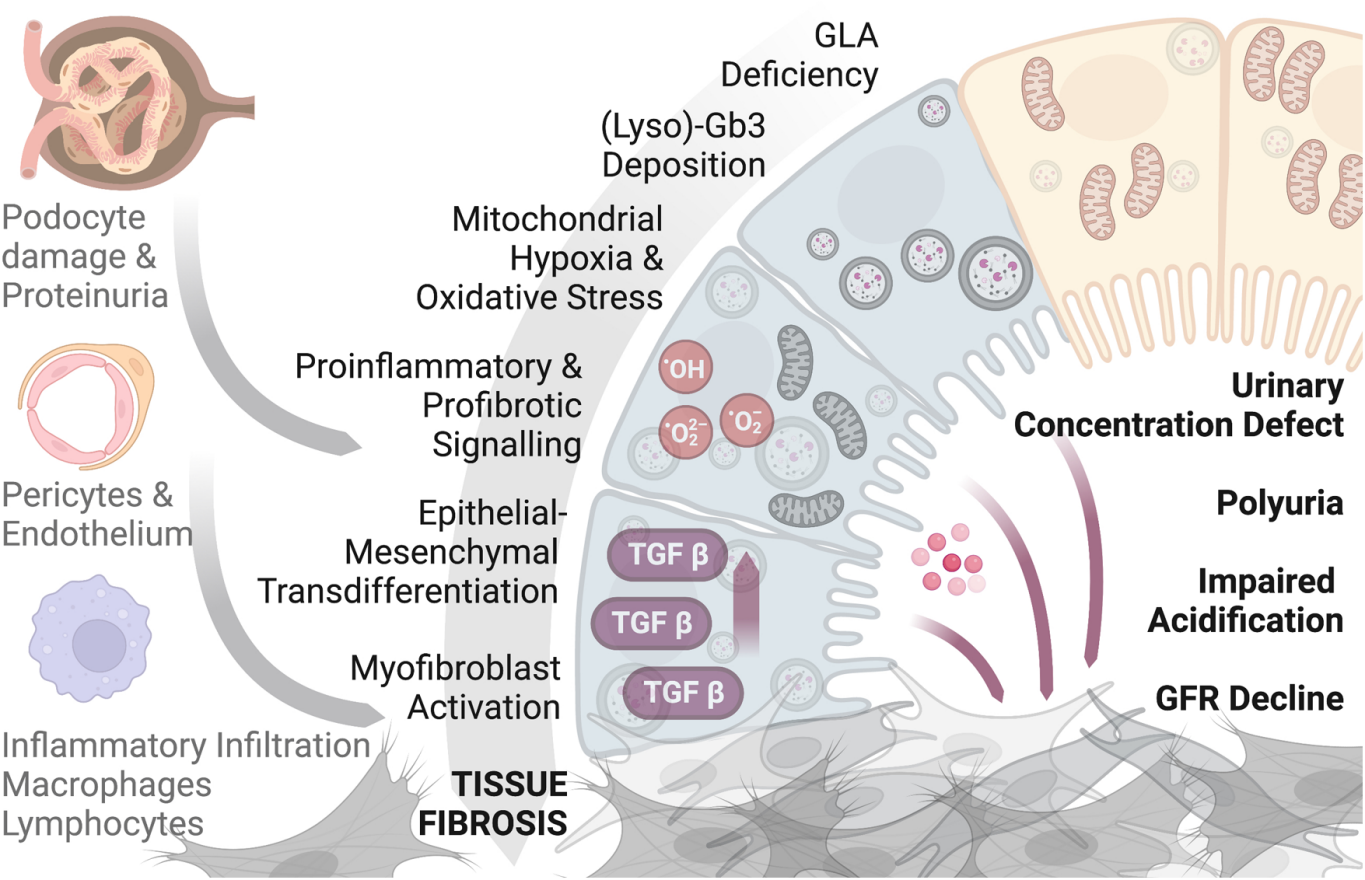
A graphical scheme showing the contribution of tubular epithelial cells and the interstitium compartment to Fabry nephropathy. Figure produced with biorender.com.

All this reported evidence demonstrates that renal fibrosis is the final result of an insult of different origin with the activation of inflammation, cellular migration, and differentiation, and an increase in the extracellular matrix. In Fabry nephropathy, these processes are present early in the tubular compartment following the primary injury of lysosomal dysfunction and a derangement of the metabolism of Gb3-LysoGb3. As evidence suggests that a mitochondrial phenotype is a partial pathogenic process, tubular cells with their energy needs and consumption may be one of the primary and early cells affected in the kidney. Hence, the contribution of tubular epithelial cells and the interstitial compartment to Fabry nephropathy could be substantial, from the starting point to a progressive and continuous development and contribution of pathological mechanisms leading to renal affection and failure (Figure 1).
